# Apolipoprotein E potently inhibits ferroptosis by blocking ferritinophagy

**DOI:** 10.1038/s41380-022-01568-w

**Published:** 2022-04-28

**Authors:** Abdel Ali Belaidi, Shashank Masaldan, Adam Southon, Pawel Kalinowski, Karla Acevedo, Ambili T. Appukuttan, Stuart Portbury, Peng Lei, Puja Agarwal, Sue E. Leurgans, Julie Schneider, Marcus Conrad, Ashley I. Bush, Scott Ayton

**Affiliations:** 1 Melbourne Dementia Research Centre, Florey Institute of Neuroscience and Mental Health, The University of Melbourne, Parkville, VIC 3052 Australia; 2 Department of Neurology and State Key Laboratory of Biotherapy, National Clinical Research Center for Geriatrics, West China Hospital, Sichuan University, Chengdu, Sichuan 610041 China; 3 Rush Alzheimer Disease Center, Rush University Medical Center, Chicago, United States; 4 Helmholtz Zentrum München, Institute of Metabolism and Cell Death, 85764 Neuherberg, Germany; 5 Pirogov Russian National Research Medical University, Laboratory of Experimental Oncology, Moscow, 117997 Russia

**Keywords:** Neuroscience, Diseases

## Abstract

Allelic variation to the *APOE* gene confers the greatest genetic risk for sporadic Alzheimer’s disease (AD). Independent of genotype, low abundance of apolipoprotein E (apoE), is characteristic of AD CSF, and predicts cognitive decline. The mechanisms underlying the genotype and apoE level risks are uncertain. Recent fluid and imaging biomarker studies have revealed an unexpected link between apoE and brain iron, which also forecasts disease progression, possibly through ferroptosis, an iron-dependent regulated cell death pathway. Here, we report that apoE is a potent inhibitor of ferroptosis (EC_50_ ≈ 10 nM; N27 neurons). We demonstrate that apoE signals to activate the PI3K/AKT pathway that then inhibits the autophagic degradation of ferritin (ferritinophagy), thus averting iron-dependent lipid peroxidation. Using postmortem inferior temporal brain cortex tissue from deceased subjects from the Rush Memory and Aging Project (MAP) (*N* = 608), we found that the association of iron with pathologically confirmed clinical Alzheimer’s disease was stronger among those with the adverse *APOE*-ε4 allele. While protection against ferroptosis did not differ between apoE isoforms in vitro, other features of ε4 carriers, such as low abundance of apoE protein and higher levels of polyunsaturated fatty acids (which fuel ferroptosis) could mediate the ε4 allele’s heighted risk of AD. These data support ferroptosis as a putative pathway to explain the major genetic risk associated with late onset AD.

## Introduction

Apolipoprotein E (*APOE* - gene; apoE - protein) is a 34 kDa lipid-transporting glycoprotein with three common isoforms conferring risk for Alzheimer’s disease (AD; risk ε2 < ε3 < ε4) [1]. The mechanism(s) for how *APOE* allelic variation imparts this risk are debated, but several physiological roles of apoE have been suggested including: regulation of synaptic function, neurogenesis, clearance of misfolded proteins, and inflammation [1]. A feature of *APOE* ε4 carriers is low cerebrospinal fluid (CSF) levels of apoE protein [2–5]. Low CSF levels of apoE is also a feature of AD irrespective of genotype, and predicts longitudinal decline [2, 3]. A putative trophic or protective function of the apoE protein, regardless of genotype, is not yet established.

We previously identified a surprising correlation between CSF levels of apoE and ferritin (a biomarker of brain iron), and we showed that CSF ferritin is ≈20% elevated in *APOE* ε4 carriers [2]. Higher brain iron measured by MRI has been reported in *APOE* ε4 carriers [6], and *APOE* ε4 genotype has been shown to influence the impact of iron on synchronized default mode network activity [7]. While a connection between a lipid transporting protein and brain iron may be unexpected, mutations in a range of lipid metabolism genes are a surprisingly common cause of Neurodegeneration with Brain Iron Accumulation (NBIA) including mutations in pantothenate kinase 2 (PANK2), phospholipase A2 group VI (PLA2G6), fatty acid 2-hydroxylase (FA2H) and CoA synthase (COASY) [8, 9].

Pro-oxidant brain iron elevation was one of the earliest described changes observed in AD brain tissue [10, 11], and has been confirmed with modern techniques [12, 13]. Recent data reveal that the burden of cortical iron in AD predicts disease progression [2, 14–18]. We also found that elevated CSF ferritin predicted accelerated cognitive decline over 7 years in pre-symptomatic *APOE* ε4 carriers and was able to discriminate stable from cognitively declining subjects [19]. In the BioFINDER cohort, higher CSF ferritin levels were associated with biomarker confirmed clinical AD that was stronger in ε4 compared to non-ε4 carriers [20].

Ferroptosis has been implicated as a mechanism linking iron burden to neurodegeneration in AD [21]. It is a regulated cell death pathway characterized by the accumulation of lethal lipid hydroperoxides resulting from tissue iron reacting with esterified polyunsaturated fatty acids (PUFAs), especially n-6 PUFAs such as arachidonic acid [22, 23], while n-3 fatty acids [24], and n-9 monounsaturated fatty acids [25] are protective. Glutathione peroxidase 4 (GPX4) is the checkpoint that functions to detoxify lipid hydroperoxides [26–28]. GPX4 requires glutathione for this activity, and depletion of glutathione (which is a feature of AD brain [29–31]), or direct inhibition of the enzyme, induces ferroptosis. In vitro, ferroptosis is classically induced by using a direct inhibitor of GPX4 such as RSL3 or an inhibitor of the cystine import channel, system xc^-^, with compounds such as erastin or glutamate. Cystine is reduced to cysteine within the cell, the rate limiting amino acid for glutathione synthesis, but, as described below, cysteine depletion is also important for iron liberation from ferritin during ferroptosis and induces a specific type of ferroptosis termed cysteine depletion-induced (CDI) ferroptosis [32].

Low cellular cysteine induces the leucine zipper transcription factor, ATF4, which upregulates DDIT4 (DNA-damage-inducible transcript 4) [33], and promotes iron release from ferritin, the major iron store of the cell, through a process termed ferritinophagy [34]. Ferritinophagy is the lysosome degradation of ferritin, targeted by the NCOA4 (Nuclear Receptor Coactivator 4) chaperone. DDIT4 is a potent inhibitor of mTOR, acting downstream of AKT by regulating the assembly of the TSC1/TSC2 (Tuberous sclerosis tumor suppressors) complex [35]. Therefore, ATF4 and DDIT4 induction (e.g. by low cysteine) promotes the release of cytosolic iron from ferritin to promote ferroptosis. This liberated iron induces lipid peroxidation in an environment with low glutathione that would otherwise act as a protective substrate for GPX4.

The mode of neurodegeneration in AD is not known. We propose that ferroptosis is a candidate mechanism for neurodegeneration in AD based on a series of clinical observations: an association of brain iron with disease progression [2, 14–19], decreased brain glutathione [29–31], and increased lipid peroxidation products (including 4-hydroxynonenal, malondialdehyde, F_2_-isoprostanes, acrolein, and depletion of long-chain PUFAs [36–41]). A role for ferroptosis in AD would be further supported if AD-related genes also impacted ferroptotic susceptibility. Here, we report that apoE potently inhibits CDI ferroptosis. We demonstrate that apoE inhibits ferritinophagy by stimulating the PI3K/AKT pathway. We provide human data from the Rush Memory and Aging Project (MAP) - a large community-based cohort study with autopsy - which reveals that the iron-dependent risk for AD was increased in those carrying the *APOE* ε4 allele. These findings support a role for apoE in suppressing ferroptosis in AD pathogenesis, and provide an explanation for why the low abundance of apoE is an independent risk factor for disease progression.

## Results

### APOE ε4 carriers are more likely to develop AD as a product of brain iron burden

Previous findings revealed that elevated CSF ferritin was associated with a faster rate of longitudinal cognitive decline in ε4 compared to non-ε4 carriers in subjects from the ADNI cohort [19]. Similarly, in the BioFINDER cohort, elevated CSF ferritin increased the risk of AD in ε4 subjects [20]. Using the MAP cohort, we recently reported that iron levels were elevated in the inferior temporal cortex of people with pathology confirmed AD [15]. Here, we expand upon this analysis by investigating the impact of *APOE* ε4 genotype on the association between iron and Alzheimer’s dementia diagnosis (confirmed by clinical and pathological characterization; 608 subjects; Table 1). We classified people as positive for the ε4 allele if they were heterozygous or homozygous carriers of ε4, while patients were classed as negative for ε4 if they had the genotype of ε2/ε2, ε2/ε3, or ε3/ε3. There were 313 non-ε4 individuals without AD, 137 non-ε4 individuals with AD, and there were 65 individuals without AD who classed as ε4, and 93 people with dementia who carried the ε4 allele.

**Table 1:** Demographics of the MAP individuals.

AD	No		Yes		No		Yes		All	
*APOE* ε4	No		No		Yes		Yes		All	
*N*	313		137		65		93		608	
Male sex: *N* (%)	98	(31.3)	34	(24.8)	26	(28.0)	26	(28.0)	176	(28.9)
Age: mean (SD)	89.5	(6.5)	92.7	(5.5)	89.7	(5.3)	89.7	(5.2)	90.1	(6.3)
Iron (μg/g tissue): mean (SD)	50.4	(16.1)	54.3	(16.0)	48.9	(12.0)	60.0	(22.7)	52.6	(17.2)
Iron (Log): mean (SD)	3.88	(0.25)	3.96	(0.27)	3.86	(0.25)	4.04	(0.30)	3.92	(0.27)

We did not additionally stratify by ε2 genotype, or ε4 homozygosity due to low numbers in either the control or AD groups. Regarding homozygous ε4, there were only 2 non-AD individuals and only 8 AD individuals. Regarding ε2/ε2 and ε2/ε3 there were only 4 and 55 non-AD individuals, and 0 and 15 AD individuals respectively. There were only 11 ε2/ε4 individuals (5 without AD, 6 with AD). Therefore, we performed our analysis using a simplified stratification of ε4, where subjects were regarded positive if they harboured at least one ε4 allele.

**Fig. 1:**
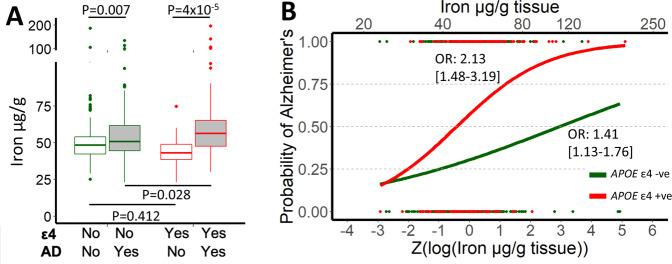
*APOE* ε4 augments the odds for AD independently conferred by brain iron. Iron levels in post-mortem inferior temporal cortex from 608 donors from the MAP study. Of these subjects, 230 had a clinical and pathological diagnosis of AD, and 378 were negative under these criteria. **A** Iron levels in subjects stratified by diagnosis and ε4 status. **B** Logistic regression curves for predicting diagnosis, stratified by ε4 status.

In a multiple regression of iron in the inferior temporal cortex (including the following covariates: age, sex, *APOE* ε4, and AD diagnosis; iron was log-transformed for normality then transformed into a Z score for ease of comparison), iron was elevated in AD subjects (β[S.E.] = 0.406 [0.087]; *P* = 5 × 10^−6^). We next tested whether there was an interaction between ε4 genotype and AD diagnosis in a revised multiple regression. Indeed, this interaction term was significant (β[S.E.] = 0.411 [0.1906]; *P* = 0.031). When we performed multiple regressions of iron in the group stratified upon *APOE* ε4 (including age, sex and diagnosis as covariates), we found that the elevation in iron in AD cases compared to controls was greater for people carrying the ε4 allele (No-AD: 48.9 μg/g; AD: 60.0 μg/g; +22.7%; β[S.E.] = 0.701 [0.165]; *P* = 4 × 10^−5^) than for those without it (No-AD: 50.6 μg/g; AD: 54.3 μg/g; +7.7%; β[S.E.] = 0.273 [0.100]; *P* = 0.007; Fig. 1A).

Next, we replicated the analysis, this time stratifying according to diagnosis and including ε4 as a predictive variable. We found that the differences in iron associated with ε4 were significant for AD subjects (No ε4: 54.3 μg/g vs ε4: 60.0 μg/g; β[S.E.] = 0.320 [0.145]; *P* = 0.028) but not controls (No ε4: 50.4 μg/g vs ε4: 48.9 μg/g; β[S.E.] = −0.105 [0.128]; *P* = 0.412). Since the level of iron in non-AD subjects with ε4 was not elevated compared to those without ε4 (indeed the mean values were lower), we cannot conclude that the presence of ε4 automatically elevates brain iron. However, these results show that higher brain iron that occurs in AD is more pronounced when carrying the ε4 genotype. Since it is unlikely that the ε4 genotype would cause iron to be higher in AD subjects specifically, we tested whether this difference in iron according to ε4 genotype and clinical status could be explained by a difference in risk attributable to iron.

To test this possibility, we performed logistic regressions using brain iron levels to predict diagnosis in those with and without *APOE* ε4. In those without ε4, elevated iron increased the odds of pathologically confirmed Alzheimer’s dementia. (OR [CI] = 1.41 [1.13–1.76]; *P* = 0.002) but to a lesser magnitude than those with the ε4 allele (OR [CI] = 2.13 [1.48–3.19]; *P* = 1 × 10^−4^). In constructing logistic regression curves of the probability of diagnosis as a function of iron from these models, it can be seen that the risk of AD in ε4 cases with low iron approximates that of non-ε4 cases with similarly low iron (Fig. 1B). With each unit elevation in brain iron, the risk for AD increases more prominently in ε4 than non-ε4 cases.

### ApoE potently opposes cysteine depletion-induced (CDI) ferroptosis

**Fig. 2:**
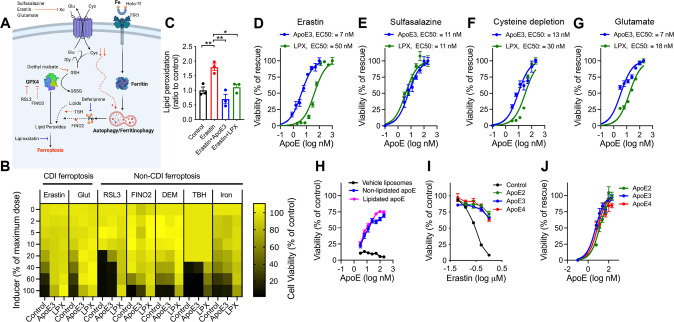
ApoE potently suppresses CDI ferroptosis. **A** schematic description of the ferroptosis pathway highlighting the inducers of ferroptosis used in this study. **B** heat map showing cell viability in response to multiple ferroptosis inducers in the absence and presence of either apoE3 (200 nM) or liproxstatin-1 (LPX, 200 nM), which was used as a standard anti-ferroptotic compound (*n* = 6). **C** Lipid peroxidation was measured using BODIPY C11 fluorescence measurement after co-incubation with erastin (0.5 µM, 16 h) and either apoE3 or LPX (200 nM) (*n* = 3). ApoE3 and LPX dose-dependent rescue from CDI ferroptosis was evaluated using erastin (**D**), sulfasalazine (**E**), cysteine depletion (**F**) and glutamate (**G**) (*n* = 4). **H** lipidation of apoE does not alter its potency to suppress erastin toxicity, but the liposomes themselves do not rescue ferroptosis (*n* = 16). ApoE2, apoE3 and apoE4 isoforms are equally effective in rescuing erastin toxicity (**I**) and show no differences in dose-dependent rescue of a single lethal dose of erastin (**J**) (*n* = 4). Data are means ± SEM and experiments were repeated at least 3 times. Data were fitted using a non-linear regression curve to determine EC50 with 95% confidence interval. P values were calculated using one-way ANOVA test corrected for multiple comparison using the Tukey test. *p < 0.05, **p < 0.01, ***p < 0.001.

That iron appears to increase odds for AD in MAP (Fig. 1) and other cohorts [2, 14–19, 42] is consistent with ferroptosis as a pathological mechanism for neurodegeneration in AD, since elevated iron lowers the threshold of toxicity of ferroptotic inducing agents. The different risk attributable to iron in ε4 and non-ε4 cases indicates that people carrying ε4 might have increased susceptibility toward ferroptosis. To test whether apoE influences ferroptosis, we assessed the impact of recombinant apoE (ε3 isoform; apoE3) upon cell death induced in N27 neuronal cells, which we previously established and characterized as a ferroptosis neuronal cell model in neurodegeneration [43]. N27 neurons were challenged with ferroptosis inducers targeting multiple arms of the ferroptosis pathway: cystine import inhibitors (erastin, sulfasalazine, glutamate), glutathione peroxidase 4 (GPX4) inhibitor (RSL3), glutathione chelator (diethyl-maleate), iron load, or direct lipid peroxidation inducers (tert-butyl hydroperoxide; TBH) (Fig. 2A). ApoE3 prevented cell death induced by erastin and glutamate but failed to protect against other ferroptosis inducers, while liproxstatin-1 (LPX), a specific ferroptosis inhibitor, prevented cell death under all conditions (Fig. 2B). Lipid peroxide accumulation is a hallmark of ferroptosis. To ensure that apoE prevented lipid peroxide accumulation in response to erastin, we monitored the effect of recombinant apoE3 on lipid peroxide accumulation using C11-BODIPY. Increased level of lipid ROS was observed after 12 h of erastin treatment, which was attenuated by recombinant apoE3 and LPX (Fig. 2C).

Erastin is a potent inhibitor of system xc-, which transports cystine into the cytosol in exchange for glutamate. Both erastin and glutamate induce intracellular cysteine depletion and impaired glutathione synthesis, ultimately leading to GPX4 inhibition and build-up of lipid peroxides. Therefore, we used multiple inducers targeting cysteine abundance to induce CDI ferroptosis and investigate apoE potency, including erastin, glutamate, the xc- inhibitor sulfasalazine, and cysteine-free media (Fig. 2D–G). ApoE exhibited robust protection against all four inducers with a potency (EC_50_ ≈ 7–13 nM) that exceeded that of the reference ferroptosis inhibitor, LPX (EC_50_ ≈ 11–50 nM), demonstrating that apoE selectively protects against CDI ferroptosis (Fig. 2D–G).

ApoE serves as a carrier that transports lipids from astrocytes to neurons and since recombinant apoE is not lipidated, we generated HDL-like apoE particles that mimic astrocyte-secreted lipidated apoE [44]. Lipidated apoE was equally effective in protecting against erastin-mediated ferroptosis (Fig. 2H), and so we continued to use recombinant non-lipidated apoE for further investigations.

We compared the potency of ε2, ε3, and ε4 isoforms and found that they provided equivalent protection against ferroptosis (Fig. 2I, J). Therefore, the clinical risk attributable to iron that is increased in *APOE* ε4 subjects (Fig. 1) cannot be explained merely by the differential potency of the protein isoform against ferroptosis. Rather, increased ferroptosis susceptibility in ε4 subjects might be due either to a change in lipid composition that promotes ferroptosis or to a decrease in apoE levels. The ratio of docosahexaenoic acid (DHA, n-3)/arachidonic acid(n-6) is decreased (favoring ferroptosis) in plasma of ε4 carriers [45, 46], which accords with findings that the half-life of DHA (assayed using [^13^C]DHA) is decreased by 30% in ε4 carriers compared to non-carriers [47]. Low levels of apoE protein are also observed in ε4 subjects [2–5], which, according to our current evidence, would derepress ferroptotic stress. While these explanations could account for why ε4 carriers have increased sensitivity to ferroptosis, the reason for why apoE in CSF is correlated with ferritin requires further elucidation.

### ApoE suppresses ferritinophagy

**Fig. 3:**
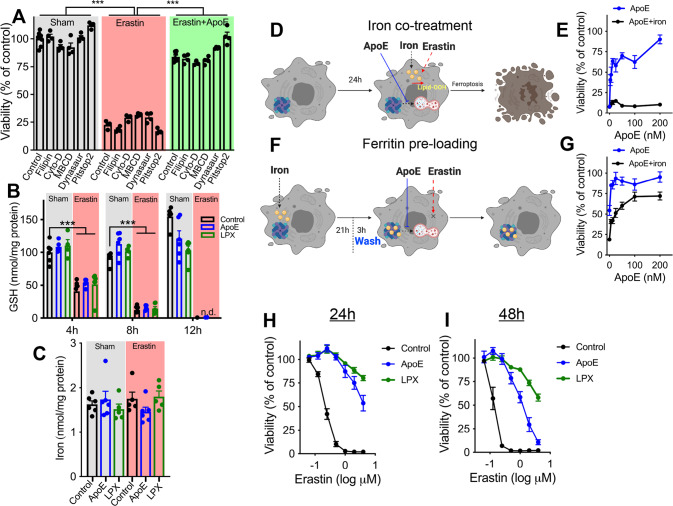
ApoE inhibition of ferroptosis is mediated by suppressing iron release from ferritin. **A** ApoE rescue of a lethal dose of erastin is not prevented by co-incubation with five different endocytosis inhibitors, shown (*n* = 4; MBCD = methyl-beta-cyclodextrin). **B** Total glutathione (GSH) was measured in N27 neurons at different time intervals following treatment with erastin (0.5 µM) in the presence of either apoE or LPX (*n* = 6). **C** Iron levels were measured in N27 neurons after exposure to erastin (0.5 µM, 16 h) in the presence of either apoE or LPX (*n* = 6). ApoE rescue of a lethal dose of erastin was abolished after incubation with iron (100 µM) (**D**, **E**) (*n* = 3), but preserved when iron was pre-loaded in ferritin prior to erastin exposure (**F**, **G**) (*n* = 5). **H**, **I** ApoE rescue of erastin toxicity diminishes over time, compared to LPX, measured by the MTT viability assay at 24 h (**H**) and 48 h (**I**) (*n* = 9). Data are means ± SEM and experiments were repeated at least 3 times. ApoE and LPX were both 200 nM. P values were calculated using one-way ANOVA test corrected for multiple comparison using the Tukey test (**A**) and Dunnett test (**B**). ***p < 0.001.

Astrocytes are the primary source of apoE in the brain and secreted apoE is subsequently internalized in neurons via various receptors. Therefore, we first investigated whether the rescue of ferroptosis by apoE involved internalization of this protein. Multiple endocytosis inhibitors failed to counteract apoE rescue of erastin-mediated toxicity (Fig. 3A and Supplementary Fig. 1A–E). Furthermore, trypsin-treatment or surface biotinylation experiments, which separate extracellularly bound proteins from proteins within the cell, revealed that apoE administered to cells predominantly localized to the plasma membrane (Supplementary Fig. 2A, B). These data imply that apoE functions extracellularly to avert ferroptosis. That apoE protected specifically against CDI ferroptotic inducers implies that apoE is not functioning by altering the lipid or iron composition of the cell in this acute experiment, since these should lead to protection against both CDI and non-CDI ferroptosis.

CDI ferroptosis differs from the direct inhibition of GPX4 in several ways. CDI ferroptosis is slower, as it is caused by a gradual depletion of glutathione, and ferritinophagy has a more prominent role in the induction mechanism [48]. Therefore, we examined whether apoE rescues ferroptosis by increasing GSH levels. Neither apoE nor LPX impacted on GSH levels in control or erastin treatment conditions (Fig. 3B and Supplementary Fig. 3), demonstrating that apoE does not rescue ferroptosis by restoring GSH. Next, we tested whether apoE influences ferroptotic iron biochemistry using different approaches. First, we measured total cellular iron levels in neurons with and without erastin intoxication, and with and without apoE or LPX treatment, and found no changes in iron in any group (Fig. 3C).

Next, we tested whether apoE impacts the mobilization of iron from ferritin that occurs as a consequence of erastin-induced ferritinophagy [48]. We reasoned that pre-incubation of (sub-toxic) iron would load iron into ferritin, and if apoE worked by preventing ferritinophagy, then iron priming would not affect apoE rescue. However, if iron was incubated simultaneously with apoE, the iron would not have time to be shunted to ferritin, but rather would be available to induce lipid peroxidation without the need for ferritinophagy, and therefore simultaneous iron co-treatment would abolish apoE protection against ferroptosis. Indeed, apoE failed to rescue neurons simultaneously treated with iron (100 µM) and erastin (Fig. 3D, E). However, apoE rescue was preserved when the same concentration of iron was administered as a pre-incubation, that permitted iron incorporation into ferritin prior to erastin treatment (Fig. 3F, G). Thus, apoE prevents erastin-induced ferritinophagy, which ordinarily causes iron to be released into the cell upon ferritin breakdown in the lysosome.

Classical autophagy inhibitors such as bafilomycin A1 and chloroquine avert ferroptosis, but their protection fatigues over time [48]. In accord with the behavior of autophagy inhibitors, apoE lost potency after long exposures (48 h) and after high doses of erastin (Fig. 3H, I and Supplementary Fig. 4A, B), despite being more potent than LPX against CDI ferroptosis when assessed at earlier timepoints (<24 h).

**Fig. 4:**
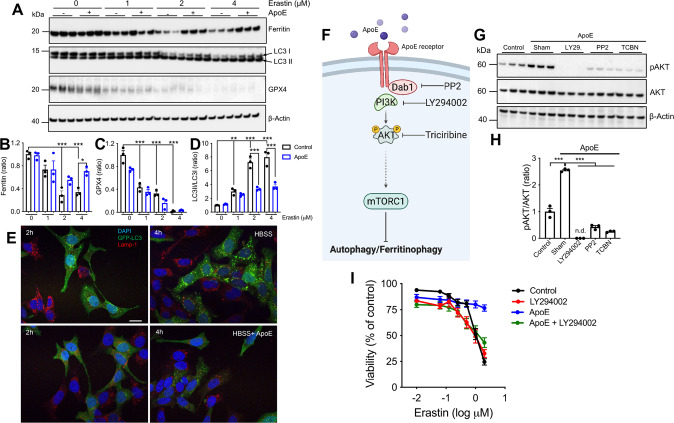
ApoE suppresses ferroptosis by inhibiting ferritin degradation through ferritinophagy. **A** Western blot of ferritin, GPX4 and LC3 levels in response to increasing doses of erastin (16 h) ± a single dose of apoE, with the quantification of ferritin, GPX4 and LC3 highlighted in (**B**), (**C**) and (**D**), respectively (*n* = 3). **E** Representative microscopy images showing the formation of LC3 puncta following autophagy induction with HBSS treatment for 2 and 4 h (top panels) and that apoE deceases LC3 puncta formation at the same intervals (bottom panels), lamp-1 and DAPI were used for visualization of lysosomes and nucleus, respectively. **F** Schematic description of the PI3K/AKT pathway that modulates ferritin degradation through the process of autophagy/ferritinophagy and the pathway inhibitors used in this study. **G**, **H** ApoE activates the PI3K/AKT pathway and induces phosphorylation of AKT, which is prevented by specific pathway inhibitors (MBCD = methyl-beta-cyclodextrin, LY29 = LY294002; *n* = 3). **I** ApoE rescue of ferroptosis is blocked by the PI3K/AKT inhibitor LY294002 (*n* = 12). Data are means ± SEM and experiments were repeated at least 3 times. A single dose of 200 nM apoE was used in all experiments. P values were calculated using one-way ANOVA test corrected for multiple comparison using the Tukey test (**B**, **C**, **D**) and Dunnett test (**H**). **p < 0.01, ***p < 0.001.

Consistent with a role in autophagy regulation, we found that apoE inhibited erastin-induced degradation of ferritin, increasing the ratio of LC3II to LC3I (characteristic of enhanced autophagic flux) [48] (Fig. 4A–D). A role for apoE in autophagy regulation extends beyond the context of ferroptosis since we found that apoE inhibited the formation of LC3 puncta (a marker of autophagy induction) when subjecting cells to amino acid withdrawal (Fig. 4E). Together, these results demonstrate that apoE protects against ferroptosis by inhibiting erastin-induced ferritinophagy.

### ApoE activates the PI3K/AKT pathway to inhibit ferritinophagy

The PI3K/AKT/mTOR signaling pathway is a major regulator of autophagy, and has been previously shown to be activated by apoE [49]. The signaling cascade involves activation of the serine/threonine protein kinase (AKT) via phosphorylation, which in turn regulates the autophagy machinery (Fig. 4F) [50]. We confirmed that apoE induced time dependent AKT phosphorylation, and that this could be blocked by specific pathway inhibitors: PP2 (inhibitor of Dab1), LY294002 (inhibitor of PI3K) and triciribine (inhibitor of AKT) (Fig. 4G, H and Supplementary Fig. 5). An exemplar inhibitor of this pathway, LY294002, abolished the ability of apoE to rescue erastin-induced ferroptosis (Fig. 4I). These data demonstrate that the anti-ferroptotic mechanism of apoE is through activation of the PI3K/AKT pathway, which inhibits ferritinophagy and prevents iron release from ferritin during ferroptosis.

## Discussion

**Fig. 5:**
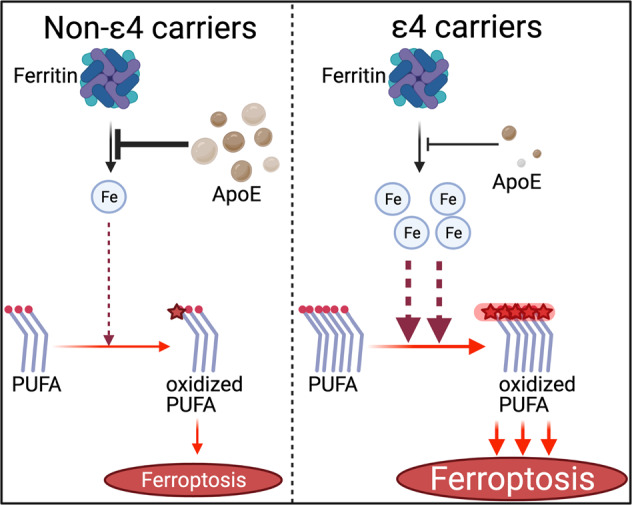
Proposed model of increased susceptibility to ferroptosis in *APOE* ε4 carriers. *APOE* ε4 carriers express high levels of oxidation-sensitive PUFAs that promote ferroptosis. Decreased levels of apoE boost ferritinophagy (reduced inhibition) and iron release from ferritin during ferroptosis, which further increases lipid peroxidation. Thus, leading to an overall increase of ferroptosis risk in *APOE* ε4 carriers.

We report that apoE potently inhibits CDI ferroptosis by suppressing ferritinophagy-dependent iron release. This study proposes an explanation for the observation that apoE protein levels, regardless of genotype, are associated with earlier cognitive decline in AD [2–5]. There was no effect of *APOE* genotype on ferroptotic inhibition, yet, in agreement with prior studies [6, 7, 19, 20], we found that iron was associated with a higher odds for AD in ε4 subjects compared to non-ε4 subjects. While the relative potency against ferroptosis cannot explain the difference in disease risk of ε4 cases, other features, such as low abundance of apoE in ε4 carriers, and altered lipid biochemistry, may explain this risk (Fig. 5).

Multiple studies have shown that apoE levels are decreased in plasma [51, 52] and CSF [2, 3, 53] of ε4 carriers. ApoE levels are reported to be allele-dose-dependent with the highest apoE levels observed in ε4 non-carriers, followed by heterozygote ε4 carriers and the lowest apoE levels in homozygote ε4 carriers [52]. Weisgraber and colleagues reported the differential stability of apoE isoforms that conforms with their abundance in vivo [54]. ApoE ε4 is more prone to unfolding than ε3 and ε2, which may promote degradation [55]. Indeed, we previously reported that zinc promotes proteolysis of apoE in an isoform-dependent manner with ε4 being most sensitive [56]. ApoE levels may also decrease in response to deposition of this protein in plaque, since, before apoE was genetically associated with AD, it was recognized as a protein enriched in plaque [57]. Regardless of the mechanism(s) that underlie lower apoE levels with the ε4 allele, our findings indicate that low apoE abundance may derepress ferroptosis in the brain. This might also explain why low CSF levels of apoE predict longitudinal decline [2, 3].

*APOE* ε4 carriers also have increased content of lipids prone to peroxidation. n-6 fatty acids (e.g. arachidonic acid and adrenic acid) phospholipids are among the most sensitive species (sensitivity increases with high unsaturation [22, 23]), while phospholipids containing n-9 (e.g. oleic acid [25]) and n-3 (e.g., DHA [24]) are resistant to ferroptosis [58]. The decreased ratio of DHA/arachidonic acid in plasma of ε4 carriers [45, 46], which accords with the shortened half-life of DHA in ε4 carriers [47], favors ferroptosis. Similarly, apoE4 altered lipid metabolism in human iPSC-derived astrocytes and led to unsaturation of fatty acids [59]. In a DHA clinical trial for AD, there was a significant cognitive improvement observed in non-ε4 carriers, but not ε4 carriers [60]. This agrees with observational studies where increased dietary intake of n-3 PUFAs (ferroptosis-resistant), and decreased dietary intake of n-6 PUFAs (ferroptosis-fueling), was associated with reduced AD risk in people without *APOE* ε4, but not with the risk allele [61, 62]. Similar results are observed for blood measures of n-3 PUFAs that are associated with protection only in non-ε4 carriers [63–65]. These clinical findings demonstrate that altered lipid biochemistry in ε4 cases leads to reduced benefit of n-3 fatty acids, which resist ferroptosis.

Brain iron levels rise during aging [9], and, if ferroptosis is more likely in ε4 subjects, this may explain why iron burden confers greater risk of disease with this genotype. While prior findings report that brain iron levels are elevated in *APOE* ε4 carriers [2, 6], here, we show that the ε4-associated change in iron (when directly measured in post-mortem cases) varies according to diagnostic group, with iron being unchanged (with a trend to decrease) in ε4 subjects without AD and higher in ε4 subjects with AD. Therefore, *APOE* may not have a direct influence on brain iron, rather, *APOE* genotype may change the risk for AD associated with iron burden. People with *APOE* ε4 are at much greater risk of developing AD, therefore those who survive without a diagnosis of AD at an advanced age must have an attribute that protects them from developing AD. We found that ε4 subjects who had low brain iron had a risk for AD equivalent to those without ε4, but this risk rapidly increased as a function of iron burden (Fig. 1B). Therefore, *APOE* ε4 may not change iron at all, rather those people with comparatively low iron remained protected from AD, but those with relatively high iron developed AD, which shifted the mean iron values higher and lower, respectively, for ε4 subjects classified as with or without AD.

A change in risk attributable to iron according to *APOE* genotype is consistent with previous findings. Cognitively normal *APOE* ε4 carriers with high CSF ferritin have increased risk of cognitive decline compared to non-ε4 carriers [19], and in another cohort, the odds ratio for CSF ferritin in predicting AD diagnosis was higher in ε4 subjects [20]. The impact of brain iron on synchronized default mode network activity was shown to be greater in ε4 carriers [7]. Post-mortem AD brain tissue of *APOE* ε4 carriers have been reported to exhibit elevated lipid peroxides [66, 67], the signature of ferroptosis.

While prior studies reveal links between *APOE* and iron or ferroptosis, our new findings reveal a direct role for the apoE protein in ferroptotic suppression, and elucidate its mechanism as the inhibition of ferritinophagy. Although initially described as a non-autophagic cell death, numerous studies have demonstrated that intact autophagy machinery is required for ferroptosis [48, 68]. Notably, ferritinophagy is an essential component of ferroptosis, causing the bolus release of iron from ferritin after selective recruitment by its cargo receptor, NCOA4, for lysosomal degradation. Gao and colleagues described the role of autophagy in CDI ferroptosis and emphasized that while genetic blockade of autophagy completely prevents ferroptosis, pharmacological inhibitors such as bafilomycin A1 and chloroquine are only effective at an earlier stage of ferroptosis [48]. Accordingly, autophagy inhibitors were effective in delaying ferroptotic cell death and lost potency 24 h after erastin treatment [48]. The apoE-mediated rescue presented here aligns with this, but apoE rescue was more persistent over time and only partial loss in potency was observed after 48 h.

A mechanistic link between apoE and ferritin was predicted by prior studies showing a strong association between these two proteins in CSF [2]. The current findings may offer an insight into this association, where apoE may prevent ferritin degradation, thereby increasing ferritin levels both in the cell, and potentially in a secreted form. Earlier studies also showed that apoE activates the PI3K/AKT signaling pathway to induce AKT phosphorylation and was blocked by the PI3K inhibitor LY294002 [49]. Our results implicate this pathway since LY294002 prevented apoE rescue of ferroptosis.

ApoE has also been shown to inhibit autophagy by acting in the nucleus to bind to “coordinated lysosomal expression and regulation” (CLEAR) DNA motifs that suppress transcription of autophagy-inducing genes [69]. *APOE* ε4 astrocytes were also shown to have lower autophagic flux compared to ε3 astrocytes [70], but it is unclear whether this was mediated by intracellular or extracellular apoE. We did not observe an isoform difference when examining the effect of apoE on autophagy and ferroptosis in neurons, but we could not exclude an isoform effect of apoE that occurs intracellularly.

These data provide a new biochemical function for apoE, and since this major AD gene functions so potently as an anti-ferroptotic agent, we hypothesize that its failure to prevent ferroptotic neurodegeneration might underlie the associated AD genetic impact. Currently, the mechanism of neurodegeneration in AD is not established, but these findings further implicate ferroptosis. Prior observations that support neuronal ferroptosis in AD are that iron is associated with cognitive decline in several longitudinal clinical cohorts [2, 14–19, 42], that glutathione is depleted in AD brain tissue [29–31], and that downstream products of lipid peroxides such as 4-hydroxynonenal, malondialdehyde, F_2_-isoprostanes, acrolein, together with depletion of long-chain PUFAs, are features of AD brain tissue [36–41]. Therefore, targeting the ferroptosis pathway becomes an avenue for therapeutic development in AD, especially in ε4 subjects.

## Methods

### The memory and aging project

The Memory and Aging Project (MAP) is an ongoing clinical-neuropathological cohort study of older adults that began in 1997 and includes Chicago residents of more than 40 retirement communities and subsidized housing [71]. At enrollment, participants were dementia free, and they agreed to undergo annual clinical neurological evaluations and brain autopsy at death. Written informed consent was obtained from all study participants, and the institutional review board of Rush University approved the study. Of the 655 MAP participants with iron quantification of the inferior temporal cortex (data and methodology reported previously [15]), we excluded those subjects who had non-Alzheimer’s dementia (i.e., dementia without Alzheimer’s disease pathologic changes; *N* = 47) which left 230 people had pathology-confirmed Alzheimer’s disease, and 378 people who died without dementia. For clinical procedures and brain neuropathology evaluation (see Supplementary Information).

### Cell culture and reagents

N27 cells, derived from E12 rat mesencephalic tissue (Cat# SCC048, Millipore) were cultured in RPMI 1640 media supplemented with 10% FBS (Bovogen biologicals), penicillin and streptomycin at 37 °C with 5% CO_2_. Erastin (Cat#S7242) and RSL3 (Cat#S8155) were purchased from Selleckchem. Liproxstatin-1 (LPX, Cat#SML1414) and MTT (Cat# M2128) were purchased from Sigma Aldrich. The endocytosis inhibitors used were: Dynasore (Cat#ab120192, Abcam), Pitstop2 (Cat#ab144650, Abcam), cytochalasin D (Cat# C8273, Sigma Aldrich), filipin (Cat#F9765, Sigma Aldrich) and methyl-beta-cyclodextrin (Cat#C4555, Sigma Aldrich). Recombinant apoE was expressed according to the protocol of Argyri and colleagues [72] and apoE lipidation was performed following the protocol of Hubin and colleagues [44] (Supplementary Methods).

### Cell Viability

Cell viability was evaluated using the MTT assay as previously described [43] or the LDH release assay according to the supplier protocol (Sigma Aldrich). Briefly, cells were cultured in 96-well plate at a density of 20,000 cells/well in growth media for 16 h. Cells were subsequently incubated with candidate compounds in growth medium for an additional 24 h. MTT was then added to the plate and absorbance was measured at 570 nm using a microplate reader (BioTek).

### Lipid peroxidation, iron and glutathione measurement

Lipid peroxidation was measured using C11-BODIPY(581/591) [22] (Supplementary Methods). Iron was measured in human brain tissues and cell extracts by inductively-coupled mass spectrometry (ICPMS) with an Ultramass 700 (Varian, Victoria, Australia) as previously described [15, 73]. Glutathione (GSH) was measured as total GSH using a colorimetric assay kit (Supplementary Methods).

### Western blot analysis

Proteins were extracted from cell pellets using RIPA buffer (50 mM Tris, 150 mM NaCl, 0.1% SDS, 0.5% sodium deoxycholate, 1% Triton X-100, containing protease inhibitors (Roche)). 15 µg aliquots of protein were separated by SDS-PAGE on 4–20% Bis-Tris protein gels (Invitrogen) and transferred to polyvinylidene difluoride (PVDF) membrane. Primary antibodies used were anti-Ferritin (cat#ab75973, Abcam), anti-ApoE (cat#AB947, Merck), anti-LC3B (cat#L7543, Sigma Aldrich), anti-GPX4 (cat#ab125066, Abcam), anti-Na/K-ATPase (cat#ab7671, Abcam), anti-AKT/pAKT (cat#4691 S/4060 S, Cell Signaling), anti-TfR1 (cat#TFR12-M, Alpha Diagnostic) and anti-β-actin (cat#A5441, Sigma). Membranes were probed with horseradish peroxidase-conjugated secondary antibodies and signal was detected using a LAS-4000 luminescence Imaging analyzer (GE Healthcare Life science). Densitometry analyses were carried out using Image J software and quantitation was normalized to β-actin levels.

### Cell surface biotinylation assay

Cell surface biotinylation of proteins was performed using the EZ-link Sulfo-NHS-SS-Biotin (Cat# 21331, ThermoFisher Scientific). N27 cells were cultivated in a 6-well tissue culture plate at a density of 250,000/well for 24 h followed by an incubation step of 16 h with apoE (200 nM) at 37 °C. After washing the cells with ice cold PBS, biotin was added to the cells, incubated for 1 h at 4 °C and cells were washed according to the protocol of the supplier. Finally, extracted proteins were analyzed with western blot using the Na/K-ATPase and β-actin as markers for membrane and soluble proteins, respectively.

### Amino acid starvation and LC3 imaging

N27 neurons overexpressing a GFP-tagged LC3 protein, were used to visualize the classical puncta distribution of LC3 in autophagosomes. pEGFP-LC3 plasmid was a gift from Toren Finkel [74] (Addgene plasmid # 24920) and was used to generate N27 cells stably expressing LC3-GFP. Amino acid starvation was performed by incubating N27 cells in HBSS and cells were subsequently fixed and immunostained using the lysosomal marker lamp1 (cat#ab24170, Abcam). Imaging was performed using a Nikon confocal laser-scanning microscope and image J software.

### Statistical analysis

All cell culture data were analyzed using Prism 9.0.1 software (Graphpad). Replicates within experiments are indicated in each figure legend and correspond to biological replicates and three independent experiments were used to ensure reliability of the reported values. Data were expressed as mean ± standard error of the mean (SEM) from independent experiments. One-way ANOVA was used to compare between groups and calculate the *p*-value and correction for multiple comparison were used as indicated in each figure legend. *p*-value <0.05 was considered statistically significant. For the cell viability data, non-linear regression analysis with a variable slope model was used to fit a curve to dose response and calculate EC50 with 95% confidence interval.

Statistical analysis of data from human samples was performed with R (version 3.3.2). We included subjects who were clinically and pathologically positive for AD (Alzheimer’s dementia) and subjects who were clinically and pathologically negative for AD. Iron data were log transformed to ensure normality, and z-transformed for unit standardization. Multiple regressions of iron were performed on the group that was stratified by [1] *APOE* ε4 allele and then [2] clinical diagnosis, and included the following covariates: age at death, sex, and either *APOE* ε4 allele or clinical AD diagnosis. Logistic regressions of diagnosis were performed in subjects that were stratified by ε4. Hypothesis tests were 2-sided.

### Supplementary information

Supplementary information
