# Supplementary material for: Apolipoprotein E potently inhibits ferroptosis by blocking ferritinophagy
Source: Mol Psychiatry. 2022 Apr 28;29(2):211–20. doi: 10.1038/s41380-022-01568-w (PMC9757994; doi:10.1038/s41380-022-01568-w)
Supplement: Supplementary file 1 — Supplementary information [file 41380_2022_1568_MOESM1_ESM.docx]

**Supplementary Methods**

Clinical evaluation procedures

All subjects underwent a uniform, structured, yearly clinical evaluation that included self-reported medical history, a neurologic examination by a trained nurse, and cognitive testing by a trained neuropsychological test technician, as previously described [[1](#_ENREF_1), [2](#_ENREF_2)]. Years of formal education, and all medications used in the prior 2 weeks were directly inspected and recorded. Medical history and neurologic examination were performed by trained nurses, and these data are reviewed annually and at time of death (blinded to pathologic diagnosis) by experienced clinicians who document evidence of stroke or Parkinsonian signs. A clinical diagnosis of AD dementia required dementia and progressive loss of episodic memory based on the criteria of the joint working group of the National Institute of Neurologic and Communicative Disorders and Stroke and the AD and Related Disorders Association (NINCDS-ADRDA) [[3](#_ENREF_3)].

Brain Neuropathology evaluation

The methods for brain autopsies and pathologic evaluations are described in detail elsewhere [[1](#_ENREF_1), [2](#_ENREF_2)]. A neuropathologist, blinded to participant ages and clinical data, determined the neuropathology diagnoses. Briefly, slabs from 1 cerebral hemisphere were placed in a -80 °C freezer and used for metal analyses. Slabs from the contralateral hemisphere were fixed in 4% paraformaldehyde, and then dissected tissue samples from brain regions were embedded in paraffin blocks, cut into 6-micron sections, and mounted onto slides.

Diffuse and neuritic amyloid plaques and neurofibrillary tangles, were identified using modified Bielschowsky silver-stained 6-micron sections in multiple cortical regions. Raw counts (greatest density in 1-mm^2^ area) of the neuritic and diffuse plaques and tangles were standardized in each region (entorhinal cortex, hippocampus, mid-temporal cortex, inferior parietal cortex, mid-frontal cortex) and averaged across regions as summary scores that reflect a global measure of Alzheimer disease pathology. Neuropathologic data was categorized as high or low based on National Institute on Aging (NIA) and the Ronald and Nancy Reagan Research Institute of the Alzheimer’s Association with criteria (Low: no or low likelihood of AD; High: intermediate and high likelihood of AD).

Lipid peroxidation measurement

Lipid peroxidation is routinely measured using a BODIPY sensor (BODIPY^TM^ 581/591 C11, Thermo Fisher Scientifics)[[4](#_ENREF_4)], a ratiometric fluorophore that responds to lipid peroxidation by a characteristic shift in its fluorescence emission peak from ~590 nm to ~510 nm, which can be measured by flow cytometry. Briefly, cells were treated with erastin (300 nM) in the absence or presence of apoE/LPX (200 nM) for 20h, followed by 1h treatment with BODIPY 581/591 C11 (125 nM) prior to harvesting with trypsin. After centrifugation, (1000rpm, 5min), cell pellet was washed twice with sorting buffer (1 mM EDTA; 25 mM HEPES; 1% FBS in 1x PBS; streptomycin/penicillin) and resuspended with 200 µl of sorting buffer containing DAPI. DAPI was added to exclude dead cells from analysis. Flow cytometry using the Cytoflex analyzer platform (Beckman Coulter) was then used to quantify the fluorescence shift.

Recombinant apoE expression and purification

Recombinant apoE was expressed according to the protocol of Argyri and colleagues [[5](#_ENREF_5)]. Briefly, the pET32a vector encoding the different apoE isoforms was used to express a thioredoxin and 6-His-tag fusion protein including a 3C-protease cleavage site between the his-tag and the apoE protein. All 3 apoE isoforms corresponding to apoE2, apoE3 and apoE4 were expressed in *E. coli* BL21 (DE3) and purified by Ni-nitrilotriacetic acid (Ni-NTA) metal affinity chromatography followed by 3C-protease cleavage (18h, 4 °C) and a final size exclusion chromatography step. ApoE protein was buffer exchanged to PBS, filtered and stored at -80 °C.

Lipidation of apoE

Lipidation of apoE was performed using recombinant apoE3 following the protocol established by Hubin and colleagues [[6](#_ENREF_6)]. Briefly, 1-palmitoyl-2-oleoyl-sn-glycero-3-phosphocholine (16:0-18:1 PC, Sigma Aldrich) and cholesterol (Sigma Aldrich) were mixed at a molar ratio of 90:5 and used to generate liposomes that mimic the lipid composition of HDL-like apoE particles. A 50 mg/ml solution of sodium cholate was titrated into the liposome solution until turbidity of the solution was cleared and recombinant apoE3 was directly added to the liposome solution at a molar ratio of 1:90:5 (ApoE: PC: cholesterol). The solution was incubated at room temperature for 1h and dialyzed against PBS for 4h at room temperature followed by a second dialysis over 72h at 4°C. After dialysis, the apoE/liposome solution was stored at 4°C until use.

**Supplementary Figures**

**
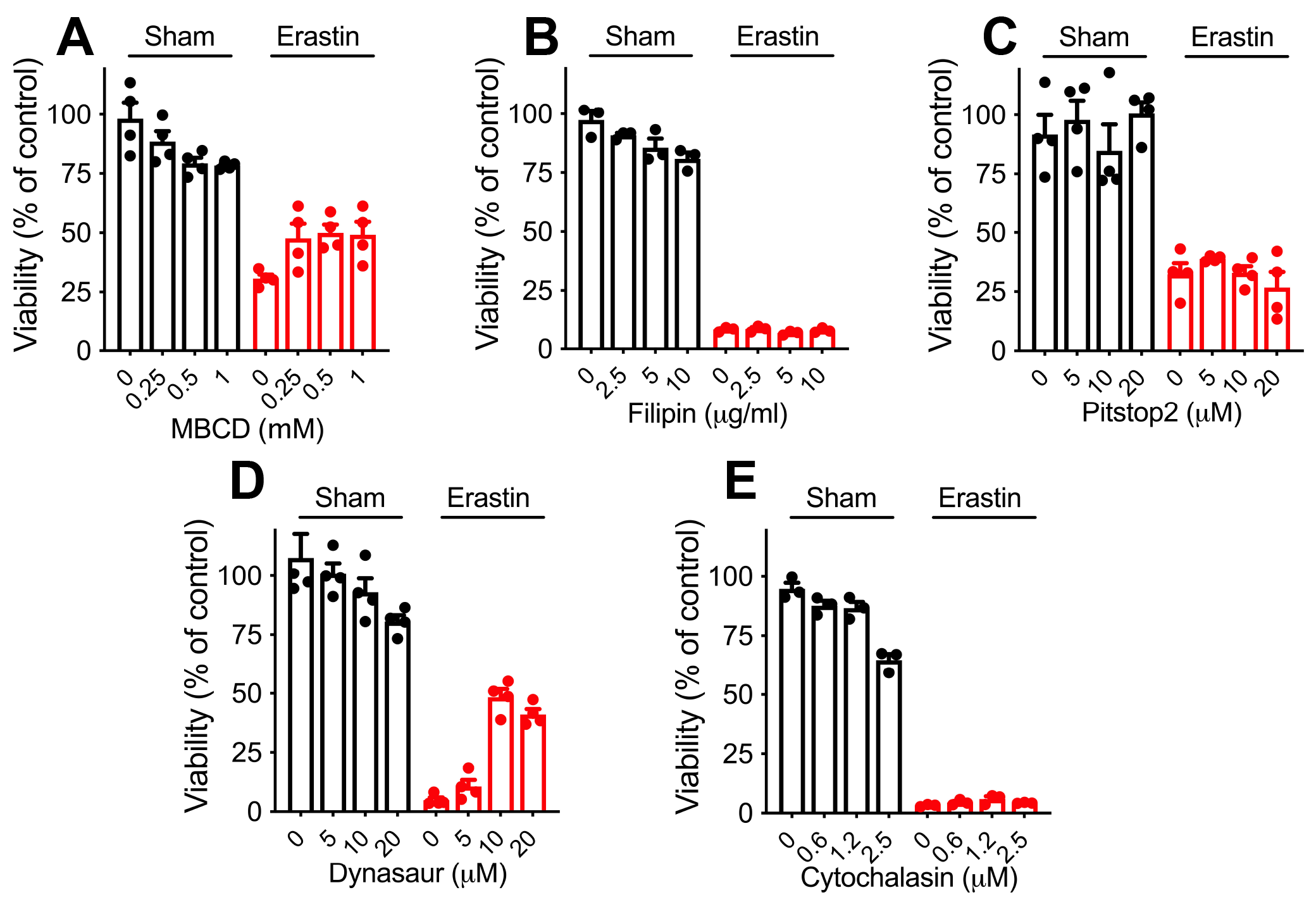
**

**Supplementary Figure 1** **Dose dependent effect of endocytosis inhibitors on viability and erastin toxicity in N27 neurons.** (**A**-**E**) N27 neurons were incubated with 5 different endocytosis inhibitors under control and erastin (0.5 µM, 16h) conditions; doses that do not cause toxicity and do not affect erastin toxicity were selected to test apoE rescue (n=4). The endocytosis inhibitors used were methyl-beta-cyclodextrin (**A**; MBCD), filipin (**B**), pitstop2 (**C**), dynasaur (**D**) and cytochalasin (**E**). Data are means ± SEM and experiments were repeated at least 3 times.

**
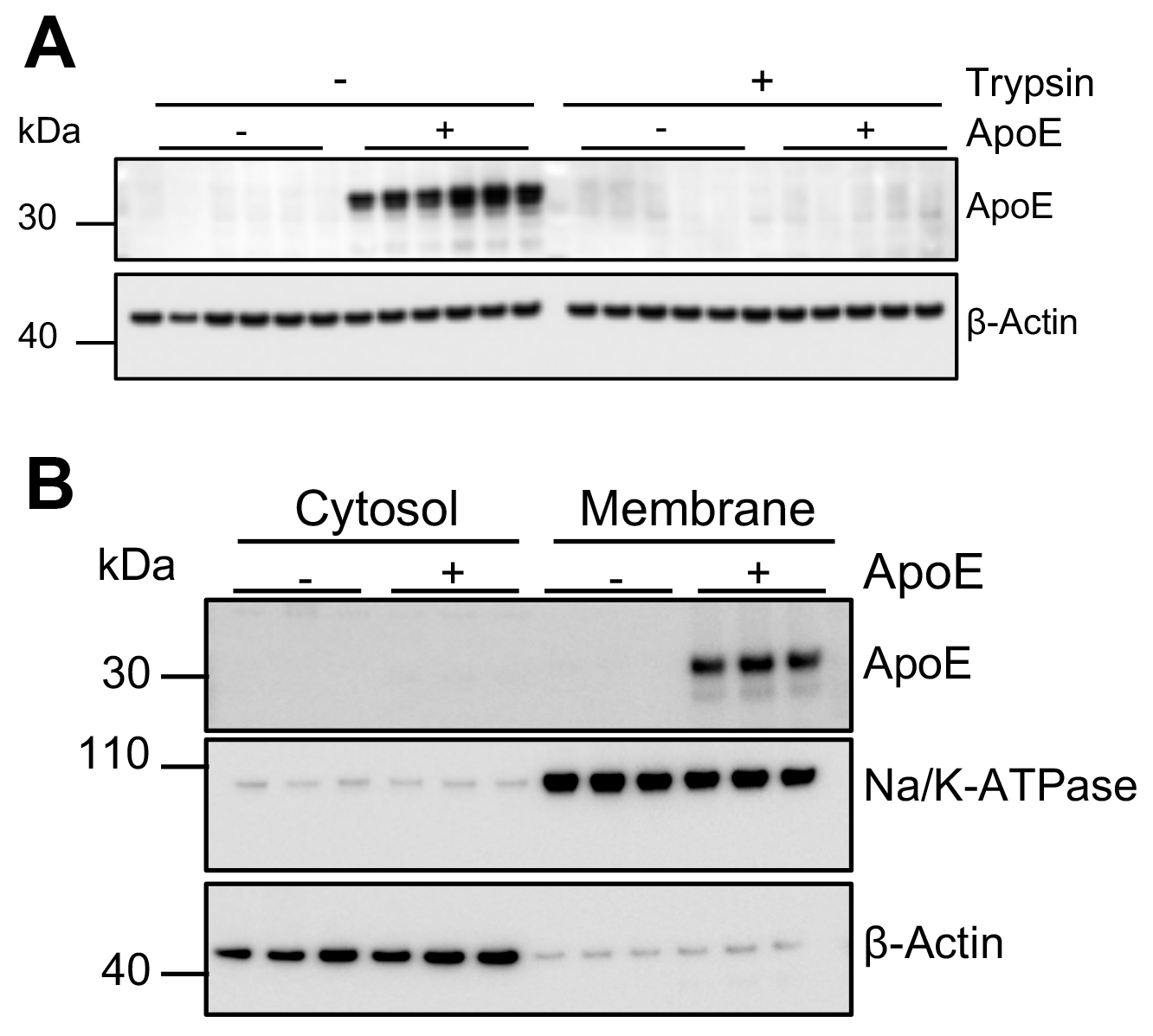
**

**Supplementary Figure 2** **ApoE is localized to the plasma membrane in N27 neurons.** (**A**) N27 neurons were incubated with apoE (200 nM, 16h) and cells were harvested either with or without trypsin treatment to digest surface exposed proteins. Western blot analysis shows that apoE is not visible after trypsin digestion, demonstrating that apoE is localized at the plasma membrane. (**B**) Western blot analysis of surface biotinylation showing that apoE is localized to the plasma membrane upon treatment of N27 neurons. Data are means ± SEM (n=3) and experiments were repeated at least 3 times.

**
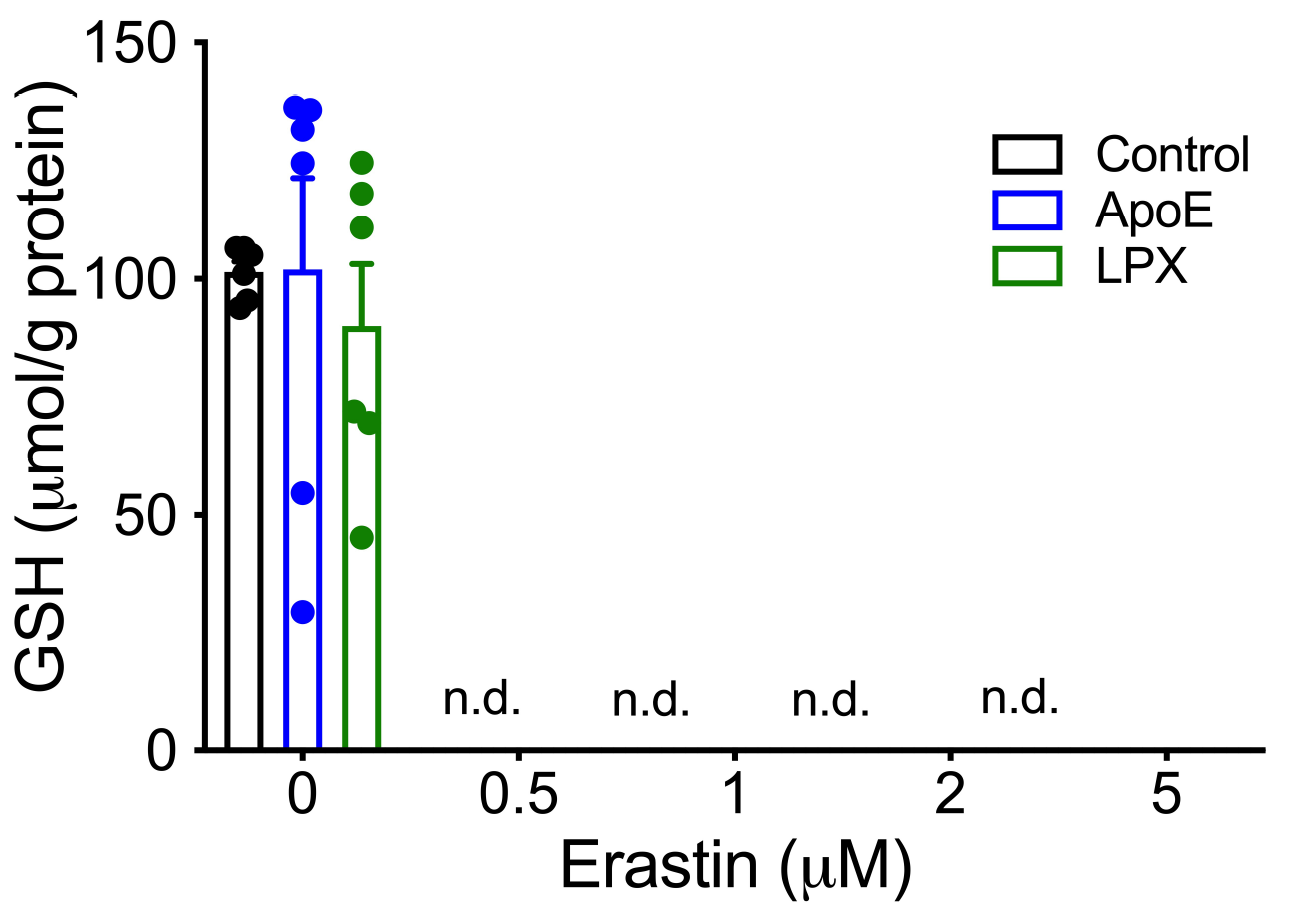
**

**Supplementary Figure 3** **ApoE and liproxstatin-1 do not alter total glutathione levels during ferroptosis.** GSH refers to total intracellular glutathione and was measured in N27 neurons under control conditions and after 12h exposure to erastin. Erastin doses ranging from 0.5 to 5 µM were effective in depleting GSH to non-detectable levels and apoE/LPX (200 nM) did not alter GSH levels under control and erastin conditions. Data are means ± SEM (n=6) and experiments were repeated at least 3 times.

**
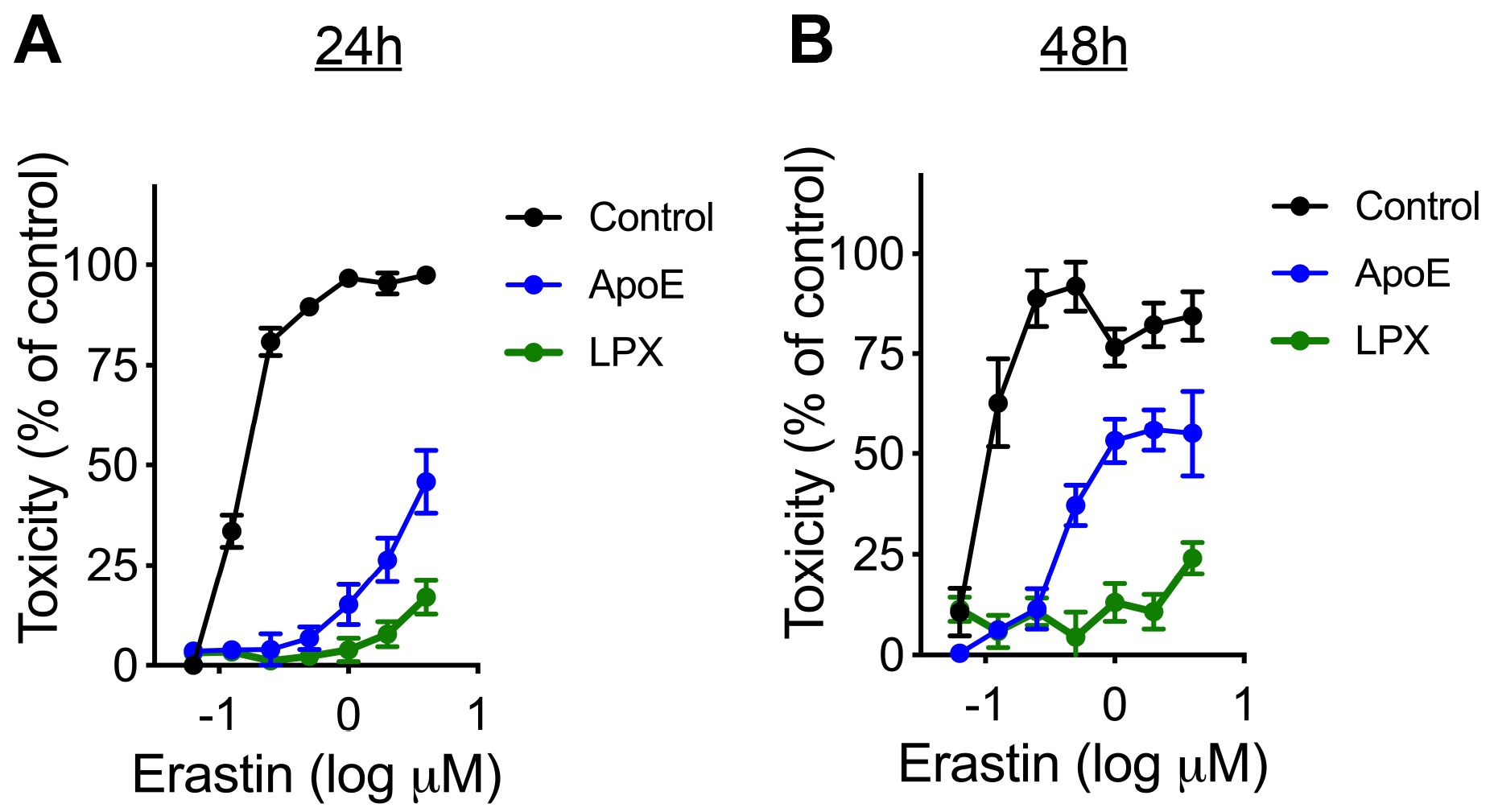
**

**Supplementary Figure 4 ApoE rescue of erastin toxicity diminishes over time.** N27 neurons were treated with a lethal dose of erastin in the presence of apoE or LPX (200 nM) and cell toxicity was measured with the LDH assay after 24h (**A**) and 48h (**B**) exposure to erastin (n=9). Data are means ± SEM and experiments were repeated at least 3 times.

**
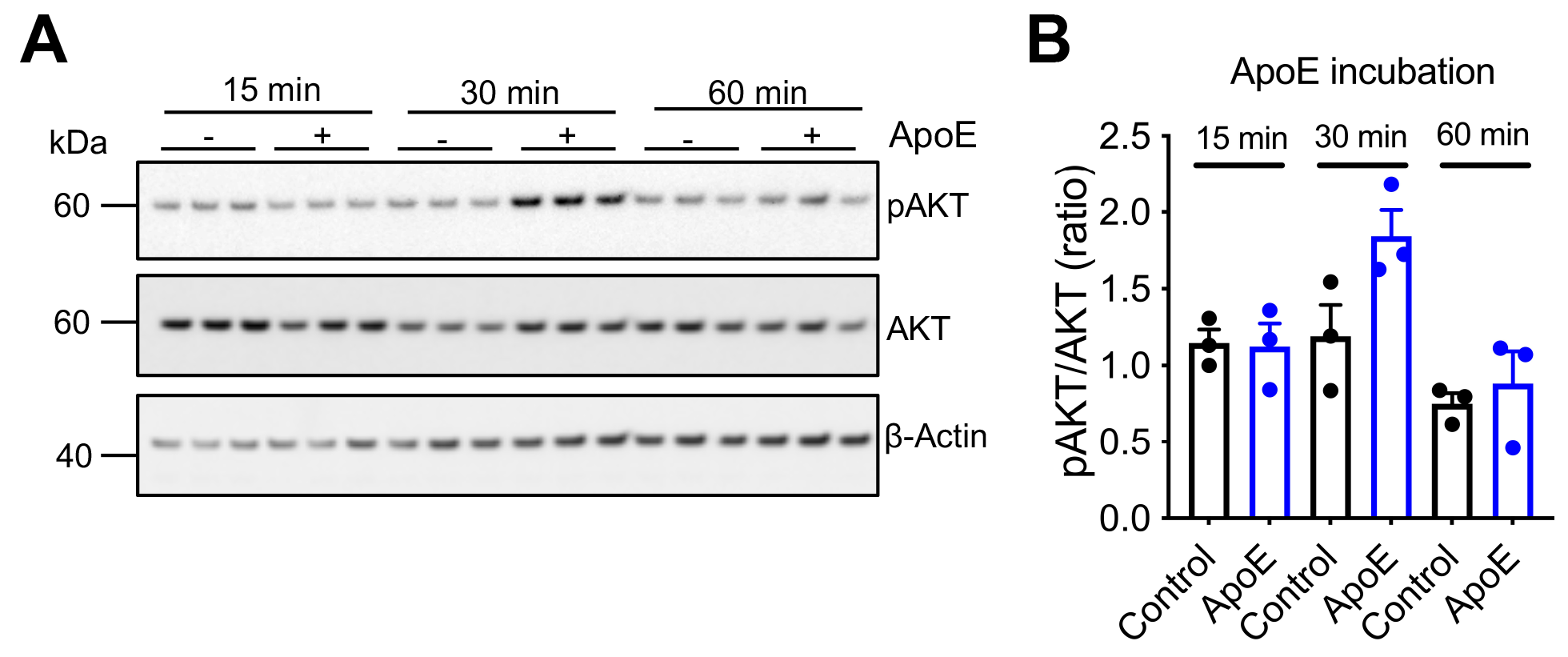
**

**Supplementary Figure 5** **ApoE induces AKT phosphorylation in a time-dependent manner.** N27 neurons were exposed to apoE (200 nM) after 15, 30 and 60 min and total AKT and phosphorylated AKT (pAKT) were analyzed per western blot (**A**) and the quantification is provided in (**B**). Data are means ± SEM (n=3) and experiments were repeated at least 3 times.

**References**

[1] Bennett DA, Schneider JA, Arvanitakis Z, Kelly JF, Aggarwal NT, Shah RC, Wilson RS (2006) Neuropathology of older persons without cognitive impairment from two community-based studies. *Neurology* **66**, 1837-1844.

[2] Ayton S, Portbury S, Kalinowski P, Agarwal P, Diouf I, Schneider JA, Morris MC, Bush AI (2021) Regional brain iron associated with deterioration in Alzheimer's disease: A large cohort study and theoretical significance. *Alzheimer’s and Dementia*.

[3] McKhann G, Drachman D, Folstein M, Katzman R, Price D, Stadlan EM (1984) Clinical diagnosis of Alzheimer's disease: report of the NINCDS-ADRDA Work Group under the auspices of Department of Health and Human Services Task Force on Alzheimer's Disease. *Neurology* **34**, 939-944.

[4] Dixon SJ, Lemberg KM, Lamprecht MR, Skouta R, Zaitsev EM, Gleason CE, Patel DN, Bauer AJ, Cantley AM, Yang WS, Morrison B, 3rd, Stockwell BR (2012) Ferroptosis: an iron-dependent form of nonapoptotic cell death. *Cell* **149**, 1060-1072.

[5] Argyri L, Skamnaki V, Stratikos E, Chroni A (2011) A simple approach for human recombinant apolipoprotein E4 expression and purification. *Protein Expr Purif* **79**, 251-257.

[6] Hubin E, Verghese PB, van Nuland N, Broersen K (2019) Apolipoprotein E associated with reconstituted high-density lipoprotein-like particles is protected from aggregation. *FEBS Lett* **593**, 1144-1153.
